# Cancer and Obesity: An Obesity Medicine Association (OMA) Clinical Practice Statement (CPS) 2022

**DOI:** 10.1016/j.obpill.2022.100026

**Published:** 2022-07-05

**Authors:** Ethan Lazarus, Harold Edward Bays

**Affiliations:** a Diplomate American Board of Obesity Medicine, Diplomate American Board of Family Medicine, President Obesity Medicine Association (2021- 2022); Delegate American Medical Association, Clinical Nutrition Center 5995 Greenwood Plaza Blvd, Ste 150, Greenwood Village, CO 80111; b Diplomate of American Board of Obesity Medicine, Medical Director/President Louisville Metabolic and Atherosclerosis Research Center, Clinical Associate Professor/University of Louisville Medical School, 3288 Illinois Avenue, Louisville, KY, 40213, USA

**Keywords:** Cancer, Clinical practice statement, Obesity, Pre-obesity

## Abstract

**Background:**

This Obesity Medicine Association (OMA) Clinical Practice Statement (CPS) provides an overview of cancer and increased body fat.

**Methods:**

The scientific information for this CPS is based upon published scientific citations, clinical perspectives of OMA authors, and peer review by the Obesity Medicine Association leadership.

**Results:**

Topics include the increased risk of cancers among patients with obesity, cancer risk factor population-attributable fractions, genetic and epigenetic links between obesity and cancer, adiposopathic and mechanistic processes accounting for increased cancer risk among patients with obesity, the role of oxidative stress, and obesity-related cancers based upon Mendelian randomization and observational studies. Other topics include nutritional and physical activity principles for patients with obesity who either have cancer or are at risk for cancer, and preventive care as it relates to cancer and obesity.

**Conclusions:**

Obesity is the second most common preventable cause of cancer and may be the most common preventable cause of cancer among nonsmokers. This Obesity Medicine Association (OMA) Clinical Practice Statement (CPS) on cancer is one of a series of OMA CPSs designed to assist clinicians in the care of patients with the disease of obesity. Patients with obesity are at greater risk of developing certain types of cancers, and treatment of obesity may influence the risk, onset, progression, and recurrence of cancer in patients with obesity.

## 1 Obesity and cancer: introduction

Beginning in 2013, the Obesity Medicine Association (OMA) created and maintained an online Adult “Obesity Algorithm” (i.e., educational slides and eBook) that underwent yearly updates by OMA authors and that was reviewed and approved annually by the OMA Board of Trustees [1]. This was followed by a similar Pediatric “Obesity Algorithm” with updates approximately every two years by OMA authors. This OMA CPS regarding cancer and obesity was derived from the 2021 OMA Adult Obesity Algorithm and is one of a series of OMA CPSs designed to assist clinicians in the care of patients with the disease of obesity.

## 2 Obesity and cancer: overview

Table 1 outlines ten takeaway messages regarding cancer and obesity. Most cancers are thought due to environmental causes [2]. In the United States in 2014, an estimated 42.0% of all incident cancers (excluding nonmelanoma skin cancers) and 45% of cancer deaths were attributable to evaluated risk factors [3]. The implication is that by avoiding environmental causes of cancer, this provides an opportunity to prevent cancer. Obesity is second only to cigarette smoking as the most common preventable cause of cancer [[3], [4], [5]]. Effective models exist to prevent obesity [[6], [7], [8], [9]]. However, inadequate resources, ineffectual education, lack of patient access, and inconsistent implementation of preventive measures, coupled with a lack of optimal coordination of science, policy, and action [10], helps account for the lack of success in population obesity prevention and why the obesity epidemic continues [11].

**Table 1:** **Ten Takeaway Messages: Obesity and Cancer.** Obesity is a leading cause of cancer. ROS: reactive oxygen species.

1.	Obesity is the second most common preventable cause of cancer and may soon overtake cigarette smoking as the most common preventable cause of cancer [15]. For nonsmokers, obesity is considered the single most common preventable cause of cancer, especially when accompanied by unhealthful nutrition and physical inactivity.
2.	Among U.S. adults, the proportion of cancers attributable to excess body weight is ∼5% for men and ∼10% for women; an increase in body weight may be contributing to an increase in cancer among young adults [16,17].
3.	No drug has an indication to treat both obesity and prevent/treat cancer.
4.	Adiposopathic consequences of obesity that promote cancer include adipose tissue cytokine production (e.g., tumor necrosis factor, interleukin-6) which may damage cellular DNA, promote gene mutations, enhance angiogenesis, promote cell proliferation, and contribute to mitochondrial and endoplasmic reticulum stress, increasing reactive oxygen species (ROS) which may further damage cellular DNA [[18], [19], [20], [21]].
5.	Adiposopathic hypoxia processes that may promote cancer include growth of adipocytes and adipose tissue beyond their vascular supply, increasing immune and angiogenic responses, accelerating the growth and progression of cancer [22]. Obesity-related sleep apnea, and its associated hypoxia, may be associated with increased cancer risk [23,24].
6.	Obesity, adiposopathy, cigarette smoking, physical inactivity, and reduced intake of antioxidant- and phytochemical-rich foods may facilitate carcinogenic oxidative stress, which is the imbalance in the creation of unstable reactive oxygen species (ROS) relative to the body's ability to detoxify these radicals [2,20,21]. ROS contributes to carcinogenesis.
7.	Additional adiposopathic adverse consequences of increased cytokine production include endothelial dysfunction, extracellular matrix abnormalities, and intravasation, which is the movement of cells from a tissue through a vessel wall into the circulation, and which is the rate limiting step of metastasis [18,22].
8.	Adiposopathic endocrine processes that promote cancer include increased cancer promoting hormones, such as estrogens, leptin, androgens in women, and growth hormones (i.e., insulin-like growth factor-1) [18]. Insulin has a high degree of homology with insulin-like growth factor, and the hyperinsulinemia of insulin resistance (often found with obesity) may play a role in tumorigenesis [25,26].
9.	Beyond their effects on body fat accumulation, foods that may increase the risk of cancer include processed meats, meats cooked at high temperature, and simple carbohydrates including sugar-sweetened beverages [27]. Among foods that may decrease the risk of cancer are whole foods rich in phytochemicals, fiber, and antioxidants (e.g., citrus fruits, cruciferous and green leafy vegetables, legumes, nuts, whole grains, and some coffees and teas) [28,29]. Physical activity may reduce proinflammatory responses and help normalize insulin and sex hormone levels, potentially reducing cancer risk.
10.	Among patients with obesity, weight reduction, as well as appropriate nutrition and physical activity may help prevent cancer, enhance the efficacy of chemotherapy for cancer, and reduce the risk of recurrent cancer [20].

**Fig. 1:**
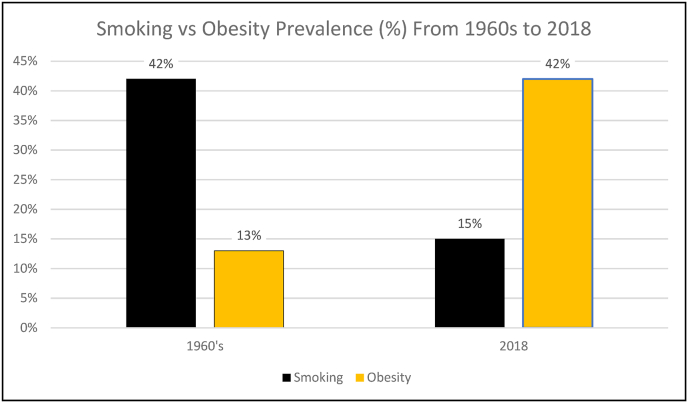
**Adult Cigarette Smoking Versus Obesity Prevalence.** Over approximately 6 decades between the 1960s to 2018, the prevalence of smoking and obesity has essentially flipped [[12], [13], [14]].

Fig. 1 demonstrates that from the 1960s up to the 2020s, the prevalence of cigarette smoking decreased as the prevalence of obesity similarly increased. Specifically, while adult cigarette smoking decreased in prevalence in the US from 42% in 1965 to 15% in 2015 [12], the prevalence of obesity in the US has flipped these percent figures, with obesity about 13% in the 1960 to 42% in 2017–2018 [13,14]. Overall, obesity may soon overtake cigarette smoking as the most common preventable cause of cancer [15].

## 3 Obesity and cancer: population-attributable fractions

Obesity increases the risk of cancers for both men and women, which include 13 cancers that account for about 40% of cancers diagnosed in the US. (Table 2) (https://www.cdc.gov/media/releases/2017/p1003-vs-cancer-obesity.html) Among U.S. adults, the proportion of cancers attributable to excess body weight is at least ∼5% for men and ∼10% for women [17,30] Table 3 lists selected risk factors for cancer and their population-attributable fractions (PAF) in promoting cancer [3]. PAFs reflect the degree that exposure to a cancer risk factor influences the occurrence of cancer. While the percent of cancers attributable to excess body weight is second in ranking to cigarette smoking, the sum of the PAF of excess body weight, alcohol consumption, physical inactivity, low fruit & vegetable intake, low fiber intake, processed meat consumption, red meat consumption and low calcium intake exceed the PAF due to cigarette smoking.

**Table 2:** **Thirteen cancers often cited as associated with obesity** (https://www.cdc.gov/media/releases/2017/p1003-vs-cancer-obesity.html).

Breast cancer (post-menopausal)
Colon and rectal cancer
Esophagus adenocarcinoma
Gallbladder cancer
Kidney cancer
Liver cancer
Meningioma
Multiple myeloma
Ovary cancer
Pancreas cancer
Stomach cancer
Thyroid cancer
Uterine cancer

**Table 3:** **Relative contributions of selected modifiable risk factors for cancer: Population-attributable fraction (PAF)** [3] Obesity is second only to cigarette smoking as the most common overall modifiable cause of cancer.

Selected Modifiable Risk factors For Cancer	Population-attributable fraction (PAF) %
All risk factors	42%
Cigarette smoking	19%
Excess body weight	8%
Alcohol	6%
Physical inactivity	3%
Low fruit & vegetable intake	2%
Low fiber consumption	1%
Processed meat consumption	1%
Red meat consumption	0.5%
Low calcium intake	0.4%
Secondhand smoking	0.4%

## 4 Obesity and cancer: genetics and epigenetics

**Fig. 2:**
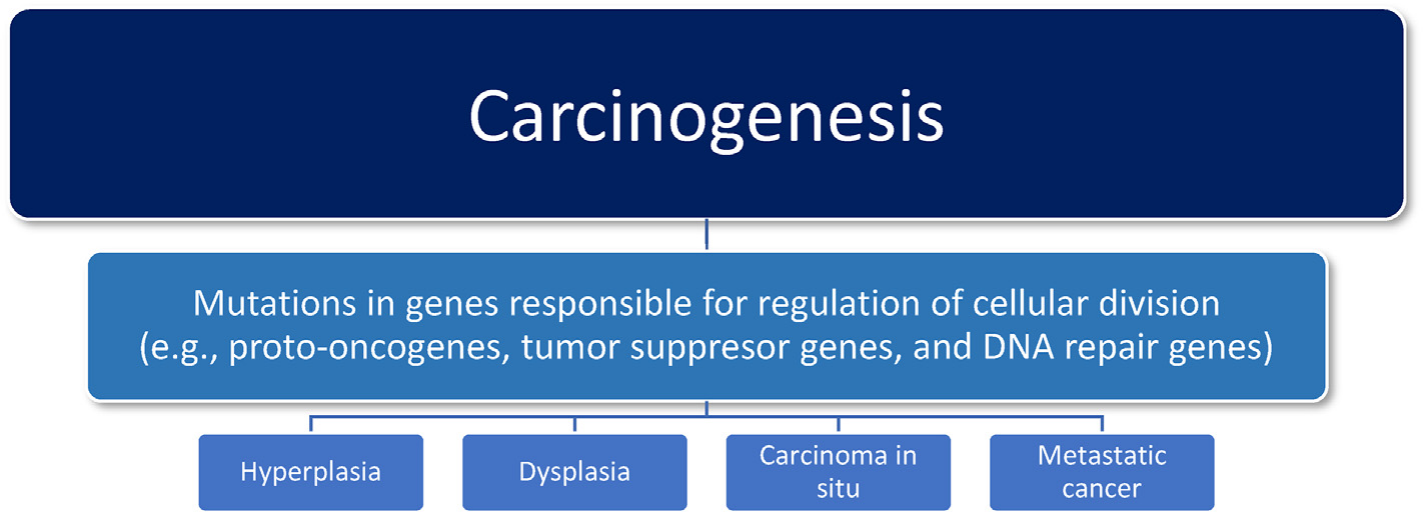
**General mechanisms of carcinogenesis.** Causes of cancer include genetic/epigenetic abnormalities, and/or environmental factors such as ultraviolet radiation, chemicals/toxins, viruses, and cigarette smoking. Obesity is also a facilitator of cancer. Disruption of orderly control of cell division (e.g., increased rate of cell division and/or impaired cell cycle arrest) can result in tissue hyperplasia (i.e., abnormal increase in cell number within a tissue or organ), dysplasia (i.e., abnormal cells within a tissue or organ), carcinoma in situ (i.e., malignant cells limited to the tissue origin), or metastasis (i.e., malignant growths at a distance from the primary tissue origin of the cancer).

Fig. 2 describes general principles regarding carcinogenesis. Cancer results from genetic disruptions of cell division, resulting in unregulated and uncontrolled tissue cell growth that enlarge locally (potentially causing adverse biomechanical consequences such as obstruction) and that potentially metastasize to other body tissues (causing peripheral non-origin tissue damage). A common finding, especially with advanced cancer, is a reduction in appetite. Among the causes of cancer cachexia includes damage to normal body tissues with subsequent inflammation, decreased hunger, increased energy expenditure, and dysmetabolism (i.e., proteolysis, lipolysis) [31,32]. One of the more identified pro-inflammatory factors associated with cancer is tumor necrosis factor (TNF), which is a cytokine that promotes tumorigenesis via stimulation of cancer cell growth, tumor angiogenesis, proliferation, invasion, and metastasis. TNF is associated with anorexia, contributing to cachexia. In fact, tumor necrosis factor was originally termed “cachectin.” [33] TNF is also produced by adipose tissue, although little evidence suggests increased TNF with obesity reduces hunger to a clinically meaningful degree. Most adipose tissue volume is composed of adipocytes, which produce TNF. However, adipose tissue also contains a stromovascular fraction (SVF), which contains preadipocytes, endothelial cells, smooth muscle cells, fibroblasts, leukocytes, and macrophages. Most of the TNF produced by adipose tissue (especially in patients with obesity) is from adipose tissue SVF macrophages [34]. TNF is illustrative of an adiposopathic link between obesity, metabolic disease, and cancer. Increased TNF with obesity impairs insulin signaling and contributes to insulin resistance and diabetes [35,36]. Increased TNF also promotes tumor cell growth, proliferation, angiogenesis, invasion and metastasis helps explain why obesity increases the risk of cancer.

Beyond environmental disruption of tissue genes responsible for regulation of cellular division, systemic genetic abnormalities may predispose patients to both obesity and cancer. In one example, utilizing genome-wide association studies (GWAS), the fat mass and obesity associated (FTO) gene was discovered in 2007 as the first gene associated with an increase in body mass index [37]. Among obesity-related genes, the FTO gene has one of the strongest links with obesity in the human population [38]. GWAS analyses have identified multiple single nucleotide polymorphisms (SNPs) of the fat mass and obesity associated (FTO) genes. The FTO gene encodes proteins participating in both adipogenesis and tumorigenesis, and is thus another link regarding obesity and cancer [39].

Obesity and obesity complications are not limited to alterations in genetic nucleotide sequencing. Alterations in gene expression can also contribute to obesity and cancer. Epigenetic dysregulation of gene expression (not involving changes in the genes themselves) may contribute to adipocyte and adipose tissue dysfunction. Regulators of gene expression include deoxynucleic acid (DNA) methylation and histone modification. Methylation of DNA at gene promoter regions typically suppresses gene transcription; histone modification can either promote or inhibit gene transcription.

Abnormalities in imprinted inheritance occur in several genetic diseases and cancer and are exemplified by the diverse genetic defects involving chromosome 15q11-q13 in Prader-Willi (PWS) [40]. Prader–Willi Syndrome (PWS) is a multisystemic genetic disorder associated with obesity caused by a lack of expression of a portion of chromosome 15. Imprinting epigenetic tags help determine which genes are to be expressed or silenced, and is a process reset during egg and sperm formation. Some genes are always silenced in the egg, and others are always silenced in the sperm. Genomic imprinting is mostly mediated by DNA methylation, usually resulting in silencing. Prader–Willi syndrome can be due to (a) non-inherited paternal 15q11–q13 gene deletion (60–70% of cases), (b) maternal uniparental disomy (two copies of the maternal gene in 25–35% of cases, thus allowing for no paternal expression of this gene), and (c) gene imprinting defects (<5%), where epigenetic silencing results in the loss of expression of the relevant paternal gene [41,42]. This is illustrative of the clinical importance of epigenetic alterations in patients with obesity.

In a more global sense, epigenetic influences on gene expression can affect obesity itself via (1) downstream effectors of environmental signals, which may involve adipogenesis and neural reward pathways; (2) abnormal global epigenetic state driving obesogenic expression patters, as might occur via mutations in epigenetic-modifier genes; (3) facilitating developmental programming, such as through early life exposures to stress, overnutrition during gestation or lactation, and chemical endocrine disrupters; and (4) transgenerational epigenetic inheritance wherein adverse epigenetic consequences of parental obesity may contribute to offspring sharing similar adverse epigenetic consequences [41]. Promoters of epigenetic alterations include obesogens (e.g., tributyltin, brominated diphenyl ether 47, polycyclic aromatic hydrocarbons, as well as endocrine disrupting chemicals such as excessive estrogens or cortisol exposure in the womb or early life), unhealthful nutrition, physical inactivity, infection, medications, toxins, and other potential environmental factors before, during, or after pregnancy (via post-partum breastfeeding, future pregnancies) [41].

**Fig. 3:**
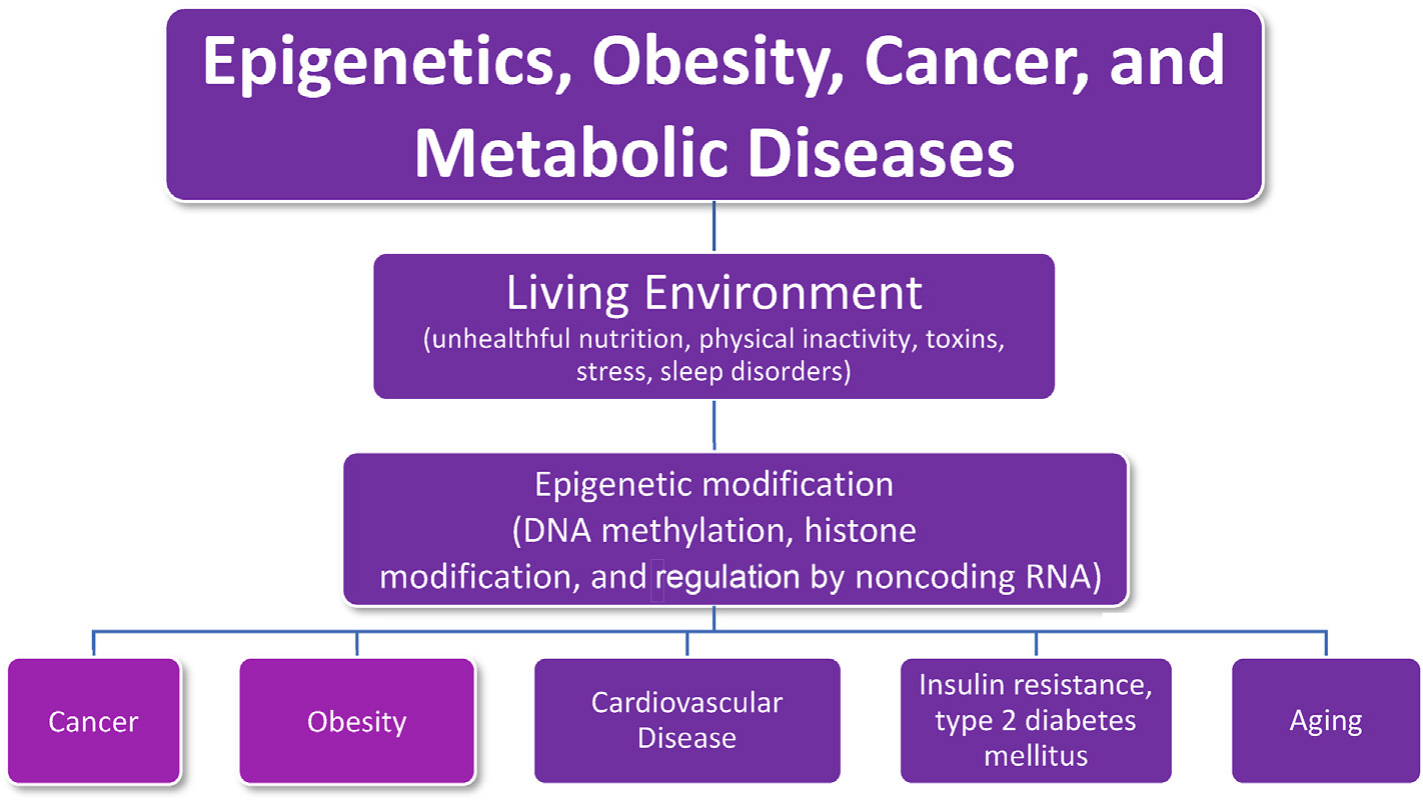
**Obesity, Epigenetics, and Cancer.** Shared obesogenic and carcinogenic living environments may contribute to epigenetic alteration in gene expression, increasing the risk of cancer, cardiovascular disease, insulin resistance, type 2 diabetes mellitus, and aging. DNA: deoxynucleic acid; RNA: ribonucleic acid.

Fig. 3 describes how obesity, cancer, and other metabolic conditions/processes share similar consequences of adverse alternations involving epigenetic gene expression. As with obesity, evidence suggests that unhealthful nutrition can adversely affect cancer epigenetics [43,44] while healthful nutrition may help reduce risk of cancer [45]. Physical activity can favorably affect obesity and epigenetic modifications [46], and routine physical activity reduces the risk for cancer and improves survival for several cancers [47]. Sleep disruption can also adversely affect obesity and epigenetic gene expression [48] and sleep disorders (commonly found with obesity) can adversely affect epigenetics and are a risk factor for cancer [49]. Anxiety can adversely affect obesity and epigenetic gene expression [50] and psychological stress increases the risk for cancer [51]. Epigenetic changes in DNA and associated chromatin proteins are increasingly considered as important mediators of the linkage between obesity and cancer [52].

## 5 Obesity and cancer: mechanisms

**Fig. 4:**
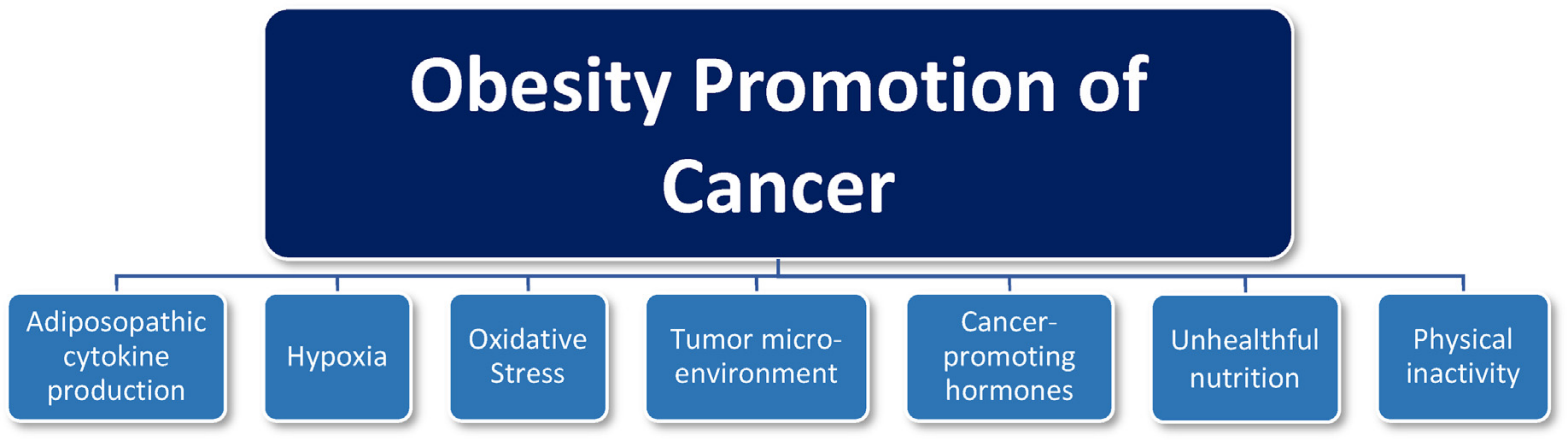
M**echanisms how obesity promotes cancer.** [3,[18], [19], [20],^22^,^23^,^30^,^43^,[65], [66], [67], [68], [69], [70], [71]] Beyond considerations of genetic and epigenetic effects, obesity may facilitate cancer via: (a) increased adiposopathic cytokine production that may damage cellular DNA, promote gene mutation, enhance angiogenesis, promote cell proliferation, contribute to endothelial dysfunction, cause extracellular matrix abnormalities and intravasation (the rate-limiting step of metastasis), and facilitate mitochondrial and endoplasmic reticulum stress, increasing the release of reactive oxygen species (ROS); examples include increased tumor necrosis factor, interleukins 6 and 1 beta, and plasminogen activator inhibitor, as well as increased leptin and resistin and decreased adiponectin; leptin activates the Janus kinase (JAK) signal transducer of activators of transcription (STAT) cellular signaling pathway, which in turn, may promote tumorigenesis, such as through promotion of cellular proliferation, angiogenesis, and anti-apoptosis, (b) expansion of adipose tissue beyond adequate vascular supply and/or increased interstitial pressure from limited expansion of extracellular matrix with relative adipose tissue hypoxia prompting the release of tumorigenic hypoxia-induced factor that accelerates blood vessel growth and thus provides oxygen and nutrients to deranged tumor cells and facilitating metastasis through neovascularization; systemic hypoxia from sleep apnea may also increase cancer risk, (c) oxidative stress through superoxide generation from NADPH oxidases, oxidative phosphorylation, glyceraldehyde auto-oxidation, protein kinase C activation, and polyol and hexosamine pathways, as well as other factors such as hyperleptinemia, low antioxidant defense, chronic inflammation, and postprandial reactive oxygen species generation, (d) alterations in the tumor microenvironment (e.g., remodeling of extracellular matrix favoring tumor development), (e) increased cancer promoting hormones (e.g., increased leptin, insulin, and insulin-like growth factors due to adiposopathic insulin resistance, increased estrogens via increased adipose tissue aromatase activity which catalyzes the conversion of steroid precursors to estrogen), and possibly promotion of cancer via an increase in androgens in women, (f) unhealthful nutrition such as consumption of saturated fats, processed/red meats, alcohol, simple carbohydrates, and trans fats, as well as reduced consumption of complex carbohydrate, fiber, fruits, vegetables, and calcium; preparation of food can also increase the risk of cancer, such as cooking meats at high temperatures, cooking with oils above their smoking point, and or repeatedly heating the same cooking oils, (g) physical inactivity may result in proinflammatory responses and carcinogenic sex hormone and insulin resistance patterns. NADPH = nicotinamide adenine dinucleotide phosphate.

Beyond excessive body fat, other factors interrelated with energy balance that increase the risk of cancer include alcohol consumption, physical inactivity, low fruit & vegetable intake, low fiber intake, processed meat consumption, red meat consumption and low calcium intake [3], as well as foods cooked in oils above their smoking temperature [53] or repeatedly heating the same cooking oils [54]. However, beyond nutritional intake alone, positive caloric balance leading to increased adiposity and dysfunctional adipose tissue can lead to endocrine and immune derangements promoting metabolic disease and cancer. Adiposopathy is defined as pathogenic adipose tissue anatomic/functional derangements, promoted by positive caloric balance in genetically and environmentally susceptible individuals, that result in adverse endocrine and immune responses that directly and/or indirectly contribute to metabolic diseases [55]. In 2014 through 2015, multiple articles were published in a four-part series in a special issue of Hormone Molecular Biology and Clinical Investigation detailed: “Adiposopathy in cancer and (cardio) metabolic diseases: an endocrine approach.” [56] Table 4 summarizes ten takeaway messages from this series of publications. Fig. 4 summarizes specific mechanisms how obesity is thought to promote cancer.

**Table 4:** **Ten Takeaway Messages: Adiposopathy and Cancer.** A 4-part series of articles published in a special issue of Hormone Molecular Biology and Clinical Investigation examined the role of adiposopathy and cancer.

1.	Cancer and (cardio) metabolic diseases are the two major ‘socio-epidemic’ illnesses in terms of prevalence and morbidity/mortality in Western industrial countries and developing countries. A major contributor to both are the multiple biological disturbances associated with adipose tissue dysfunction (i.e., adiposopathy) [57].
2.	Fat tissue is a paramount coordinator of key biological processes such as differentiation, tissue regeneration, cell growth, apoptosis, reproduction, immunity, inflammation, angiogenesis, metabolism, and thermoregulation. Adipose tissue dysfunction can lead or contribute to multiple pathologies such as obesity, type 2 diabetes mellitus, dyslipidemia, hypertension, atherosclerosis, metabolic syndrome, cognitive alteration, and/or cancer [56].
3.	Expansion of adipose tissue is achieved when proliferation and differentiation of new fat cells exceed fat cell death (apoptosis). Beyond storing excess energy, expansion of adipose tissue functions to sequester cytotoxic free fatty acids and lipid metabolites. Limitations in the proliferation and differentiation of peripheral subcutaneous adipose tissue and increase in fat deposition in the intraperitoneal area (visceral fat) promotes metabolic and vascular disturbances [58] that reflect a more carcinogenic clinical profile.
4.	Proposed mechanisms that link obesity/adiposity to high cancer risk and mortality include obesity-related insulin resistance, hyperinsulinemia, sustained hyperglycemia, glucose intolerance, oxidative stress, inflammation and/or adipocytokine production [59].
5.	In addition to increased release of pro-inflammatory factors, obesity is associated with a decrease in anti-inflammatory factors, such as a reduction in adiponectin. Adiponectin is a multimeric protein of the white adipose tissue presenting anti-inflammatory, insulin-sensitizing, anti-atherogenic, cardioprotective, and anti-neoplastic properties. Its anti-neoplastic actions are manifested via two mechanisms: (i) direct action on tumor cells by enhancing receptor-mediated signaling pathways and (ii) indirect action by regulating inflammatory responses, influencing cancer angiogenesis, and modulating insulin sensitivity at the target tissue site [60].
6.	Beyond the systemic tumor-promoting impact of adiposopathy (“sick fat”), local adipose tissue paracrine functions may lead to phenotypic and/or functional modifications of both adipocytes and cancer cells, as well as alterations in the extracellular matrix. In addition to de-differentiation processes, adiposopathy promotes cancer cell aggressiveness through promoting cancer proliferation, migration, and invasion, accompanied by tissue remodeling. In total, obesity allows for a locally permissive environment for cancer cells [61].
7.	Obesity is an important risk factor for several cancer types, such as colorectal, hepatic, esophageal, kidney, post-menopausal breast, and endometrial. Obesity or adiposopathy is also a poor prognostic factor for relapse-free survival and chemotherapy resistance [62].
8.	Breast cancer was one of the first cancer types where a positive correlation was found between obesity and breast cancer incidence and prognosis in post-menopausal women. Research supports that “sick” adipose tissue (adiposopathy) paracrine and endocrine functions play a role of adipose tissue in breast cancer initiation and progression – and may serve as an “ally of the enemy” and “collaborator” in human breast cancer [63].
9.	The adiposopathic hormone secretions most relevant to colorectal tumorigenesis include adiponectin, leptin, resistin, and ghrelin, which are involved in cell growth and proliferation, as well as tumor angiogenesis. Adiposopathy may facilitate an unfavorable adipokine profile, with an increase in pro-inflammatory and pro-cancerous immune response and a decrease in anti-inflammatory and anti-cancerous activity, potentially worsening colorectal cancer prognosis [64].
10.	Epidemiological studies demonstrate that racial and ethnic populations (e.g., non-Caucasian females, African-American females) with higher rates of obesity have higher mortality from endocrine-responsive cancers [62].

## 6 Obesity and cancer: oxidation and oxidative stress

One of the shared pathways in the multifactorial promotion of cancer by obesity is increased oxidative stress caused by reactive oxygen species [72]. Oxidation occurs when a substance gives away electrons and oxygen gains electrons; it is the opposite of reduction where a substance gains electrons and where oxygen loses electrons. From a health perspective, oxidation can be healthful (i.e., instances of natural apoptosis or release of free radicals by immune cells to fight pathogens) or harmful (i.e., promotion of cancer and/or cardiometabolic diseases) [73]. Common examples of the natural consequences of oxidation include:

• Oxidation of iron is rust or corrosion due to formation of iron oxide
• Oxidation of therapeutic fish oils causes rancidity due to hydroperoxides that can be mitigated by incorporation of vitamin E (an antioxidant)
• Oxidation of a cut apple causes it to turn brown due to oxidation of apple polyphenols

As previously noted, some people have inherited, genetic predisposition to cancer. However, as also previously noted, most human cancers are not due to genetic inheritance. Rather, as much as 90% of cancers may be due to environmental causes (e.g., smoking, obesity, UV radiation, toxins, infections, and chemical exposure), that often involve oxidative stress and oxidative tissue damage that promote cancer initiation and progression [2]. Oxidative stress is caused by an imbalance in the redox status of the body, resulting in the increased release of free radicals that damage body tissues. Reactive oxygen species (ROS) contain unstable free radicals (i.e., molecules with unpaired electrons) that cause damage to DNA, RNA, proteins, as well as contribute to cellular death. ROS affect signaling pathways such as growth factors and mitogenic pathways, and thus helps control cell proliferation that stimulates the uncontrolled growth of cells (Fig. 2). Increased oxidative stress caused by reactive species allow for angiogenesis and metastasis of cancer cells. Thus, oxidative stress plays a critical role in the initiation and progression of various types of cancers [72].

**Fig. 5:**
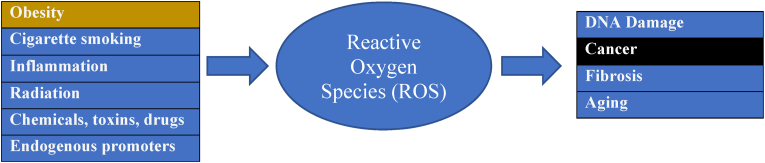
**Contributors to reactive oxygen species (ROS) creation (i.e., obesity) and adverse clinical consequences (i.e., cancer).** Shown are factors that contribute to the creation of unstable reactive oxygen species and their potential negative impacts on health. Factors include exogenous/environmental factors as well as endogenous factors such as NADPH oxidase in the cellular plasma membranes, myeloperoxidases in the lysosome, and electronic transport in the mitochondria [78]. NADPH: nicotinamide adenine dinucleotide phosphate.

Obesity, adiposopathy, smoking, and physical inactivity promote “oxidative stress,” which is the imbalance in the creation of unstable reactive oxygen species (ROS), relative to the body's ability to detoxify these radicals (i.e., via “anti-oxidants”) [21,74]. ROS (e.g., superoxide) oxidize and damage DNA and contribute to cancer and aging [21,[74], [75], [76]]. Antioxidants found in unprocessed foods (e.g., plant foods, fruits, vegetables, whole grains, and nuts) may counterbalance oxidation [21,29,73,77]. Fig. 5 shows contributors to oxidative stress and the creation of unstable ROS as well as potential adverse health consequences.

## 7 Obesity and risk of specific cancers: observational and mendelian randomization data

**Fig. 6:**
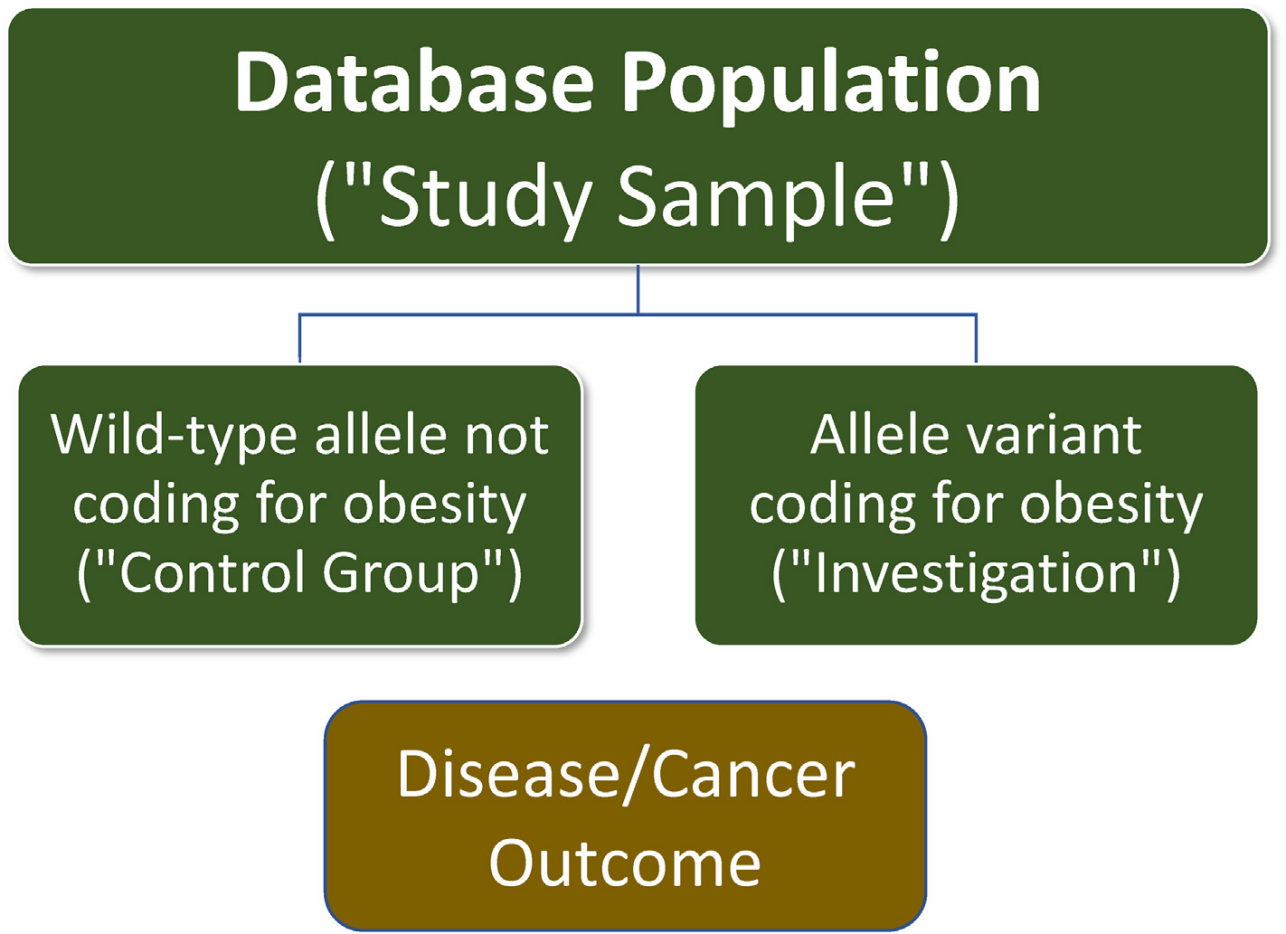
**Mendelian randomization studies help identify obesity-related cancers.** Mendelian randomization studies are analogous to conduct of a randomized clinical trial. The database population includes naturally randomized wild-type alleles (non-mutated alleles) and variant alleles, which represent the population being studied. In this conceptualization, patients with non-mutated alleles would be the control group; patients with allele variants coding for obesity would represent the exposure being investigated (i.e., similar to a clinical trial evaluating a toxin, drug, or treatment). The clinical outcomes of a patient having a variant allele coding for obesity can be compared to an individual with a wild-type allele, to determine the prevalence of an adverse consequence to obesity (i.e., cancer). If within a database, patients having an allele variant associated with obesity are found to have a higher rate of a certain kind of cancer compared to wild-type alleles, then this may suggest that obesity is a potential contributor to that type of cancer.

Population attributable fraction analyses are most often based upon observational data (Table 3), and thus susceptible to confounders [79]. It is difficult to conduct definitive long-term prospective clinical trials to assess the effect of obesity on chronic diseases such as cancer, because of the logistical challenges in randomization, because any effect obesity may take many years or decades to manifest, and because of ethical considerations. An alternative to an observational study or a randomized clinical trial is a Mendelian randomization study. A Mendelian randomization approach is a method of using known genetic variations and their function to provide inferences regarding the potential causal effect of a phenotypic expression of a gene variant. GWAS rely upon a large searchable public database of information about patient genotype and associated phenotype (e.g., Online Mendelian Inheritance in Man) [80], and involve tens of thousands of common genetic variants (i.e., most often single nucleotide polymorphisms or SNPs) associated with hundreds of complex traits (e.g., obesity, diabetes, cancer) [81,82]. A gene is a sequence of nucleotides in DNA that encodes for the synthesis of a gene product (e.g., protein). While gene size are highly variable, some estimate the average gene size is approximately 1000 nucleotide pairs, with SNPs occurring approximately once in every 1000 nucleotides [83]. An allele is a version of a gene. Mendelian randomization is an analysis utilizing cross-sectional information from patient databases to compare disease outcome effects of a “naturally randomized” distribution of wild-type (“normal” or “control”) alleles versus variant alleles within a population. This is analogous clinical trials that compare the disease outcomes of an intervention versus a control group. (See Fig. 6).

Utilization of Mendelian randomization is best considered complimentary to other data used to assess potential causality. While they may infer causality, Mendelian randomization studies alone report associations (not necessarily causally), assume the clinical outcome is only due to exposure to the phenotypic expression, and assume the phenotypic expression does not share a common cause with the clinical outcome [84,85]. Clinically, Mendelian randomization studies have helped confirm that obesity increases the risk of diabetes, diabetes-related eye disease, dyslipidemia, gout, sleep disorder, arthritis, and myocardial infarction [86]. Mendelian randomization studies further support adult obesity as potentially causing several cancers, including esophageal adenocarcinoma and cancers of the colorectum, endometrium, ovary, kidney, and pancreas [86]. With some exceptions, cancers with the greatest association to obesity are organs from the gastrointestinal system, genitourinary system, and hormone sensitive tissues. Table 5 describes the observational study strength of evidence of the association between obesity and various cancers [87,88], as well as supportive evidence from Mendelian randomization studies.

**Table 5:** **Cancers associated with obesity based upon epidemiologic/observational and Mendelian randomization studies.** The reported associations and strength of association of obesity to specific cancers vary depending on the reported analyses. Shown are the strength of evidence of the association based upon observational studies, [[88], [89], [90], [91], [92], [93]] as well as support from Mendelian Randomization [85,[94], [95], [96], [97]]. With rare exceptions (i.e., meningioma), cancers having the both epidemiologic/observational and Mendelian evidence of obesity-related causality are cancers of organs associated with the gastrointestinal system, genitourinary system, and sex hormone sensitive tissues. Because of the data considered, the cancers listed in this table may not be consistent with the 13 commonly cited increased adiposity-related cancers listed in Table 2. This table is intended to be an evolving one, with future analyses and updates altering the table conclusions (with the same principle likely to apply to the 13 cancers listed in Table 2).

CANCERS OFTEN REPORTED TO BE ASSOCIATED WITH OBESITY		
Cancer	Strength of Evidence based on epidemiologic observational studies	Mendelian Randomization Support
Biliary tract cancer	Inadequate	Not sufficient evidence
Bladder cancer	Yes to Inadequate	Yes
Brain cancer (i.e., meningiomas)	Sufficient	Yes to No
Breast cancer female (i.e., postmenopausal)	Sufficient	Yes (or not sufficient power)
Cervical cancer	Cervical cancer risk may be increased because of underdiagnosis of cervical precancer	Yes
Colorectal cancer	Sufficient	Yes
Endometrial/uterine cancer	Sufficient	Yes
Esophageal cancer (i.e., adenocarcinoma, not squamous cell carcinoma)	Sufficient	Yes
Gallbladder cancer	Sufficient	Yes (or not sufficient power)
Head and neck squamous cell cancer	Inconsistent	No
Kidney/renal cancer	Sufficient	Yes
Leukemia	Limited	No
Liver cancer	Sufficient	Yes
Lung cancer	Inadequate	Yes
Multiple myeloma	Sufficient	No or not sufficient power
Non-Hodgkin lymphoma (i.e., diffuse large B-cell lymphoma)	Limited	No
Ovary cancer	Sufficient	Yes
Pancreatic cancer	Sufficient	Yes
Prostate cancer (fatal; prognosis is worse, not necessarily increased risk)	Limited	Not sufficient power
Skin cancer (i.e., melanoma)	Inadequate	No
Stomach cancer (i.e., cardia)	Sufficient	Yes (or not sufficient power)
Testicular cancer	Inadequate	No
Thyroid cancer	Sufficient	No (a potential association does exist between type 2 diabetes mellitus and thyroid cancer)

## 8 Obesity and cancer: nutrition

Individuals, especially those living with obesity and/or those living with cancer, benefit from healthful nutrition and routine physical activity. A priority is that patients avoid cancer-promoting foods (e.g., processed meats and alcohol) [98] and other carcinogenic exposures, while favoring the intake of healthful foods. While the data is not always consistent [99], foods thought most beneficial in reducing the risk of cancer include whole foods (with limited processing) containing fiber, phytochemicals, and antioxidants [29,75,100]. (Fig. 4 and Table 6) Antioxidants derived from healthful native whole foods are most associated with reduced cancer risk, compared to concentrated antioxidants supplied in vitamin pills or supplements. Vitamin pills or supplements are sometimes associated with increased cancer risk and increased cancer mortality [73,101] The United States Preventive Services Task Force (USPSTF) recommends against the use of beta carotene or vitamin E supplements for the prevention of cancer or cardiovascular disease [102].

**Table 6:** **Foods Thought Beneficial in Cancer Prevention.** [29,29,100,100,109,110] Foods often described as beneficial in cancer prevention include fruits, berries, vegetables (especially cruciferous and green leafy vegetables), legumes, nuts, high fiber whole grain foods, and some coffees and teas (i.e., without high-calorie additives). In general, whole food (with limited processing) plant-based dietary intake not only reduces the risk of ischemic heart disease, but may also reduce the risk of cancer [111].

Fruits	Vegetables	Other
Citrus fruits such as oranges, tangerines, grapefruits, lemons, and limes.	Cruciferous and green leafy vegetables such as garlic, carrots, spinach, and broccoli	Legumes such as dry beans and peas
Apples, cherries, grapes, grapefruit, tomatoes, and squash		Nuts such as walnuts
Berries such as strawberries, raspberries, blackberries, cranberries, and blueberries		High fiber whole grains
		Some coffees and teas (without high-calorie additives)

Reduced consumption of complex carbohydrates, fiber, fruits, vegetables, and dietary calcium is a risk factor for cancer (Table 3). Increased consumption of saturated fats, processed/red meats, alcohol, simple carbohydrates, and trans fats can also increase cancer risk (Fig. 4). Ultra-processed meats of particular concern regarding increased cancer risk include many types of bacon, sausages, lunch meats, and hot dogs [28]. Cooking styles can also increase intake of carcinogens:

• Cooking meats at higher temperatures (over 300°) may result in the formation of carcinogenic compounds [28,[103], [104], [105]].
• More smoke is produced during grilling, which is linked to certain cancers [106,107].
• Cooking oils above their smoking points increases cancer risk [53].
• Repeatedly reheating and cooking with the same cooling oil [43,108].

## 9 Obesity and cancer: physical activity

**Fig. 7:**
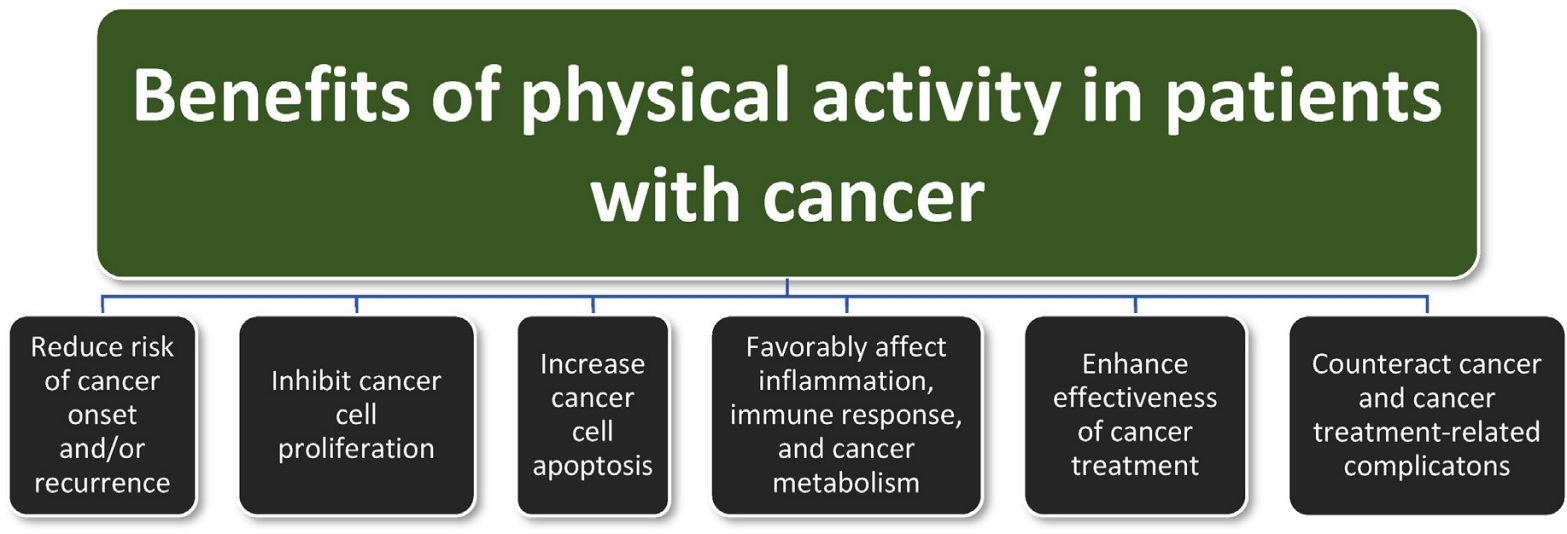
**Physical activity and physical exercise benefits in patients with cancer.** [112,113,116,117] Among the benefits of physical activity in patients with obesity are beneficial effects regarding patients at risk for cancer, or who have cancer.

Physical exercise is a component of physical activity that can enhance motivation to adopt favorable lifestyle behaviors, improve aerobic fitness, improve physical function, control fatigue, and enhance quality of life [112]. Encouraging patients to achieve physical activity goals is an important pillar to obesity management [113]. As with obesity, routine physical activity and exercise is also an important intervention to prevent and treat cancer ant its complications. Fig. 7 summarizes how increased physical activity may:

• Reduce the risk of cancer (e.g., reduced risk of breast, colorectal, gastric, endometrial, esophageal, kidney, and lung cancer), with many of these being the same cancers whose risk is increased with obesity [112]. (Table 2 and 5)
• Inhibit cancer cell proliferation and induce apoptosis [112].
• Favorably affect the surrounding immune environment (i.e., favorably affect natural killer cells, cytokines, and T-cells) and cancer metabolism [i.e. reduce angiogenic vascular endothelial growth factor (VEGF), reduce lactate dehydrogenase levels (reducing lactate levels in cells, with increased lactate dehydrogenase levels suggesting a poor prognosis for malignant tumors), and increase adenosine monophosphate-activated protein kinase (AMPK) which may oppose tumor progression and induce apoptosis of cancer cells] [112].
• Enhance the effectiveness of cancer therapies (i.e., chemotherapy) with a potential to reduce cancer recurrence and mortality among some cancer patients [114].
• May effectively treat cancer and cancer treatment-related complications such as cancer-related fatigue, osteopenia, muscular weakness, cardiac toxicity, cognitive decline, depression, and deteriorating functional capacity [112,115].

## 10 Obesity and cancer: weight reduction

While unintentional weight reduction suggests poor cancer prognosis, intentional weight reduction may be beneficial for patients with obesity/pre-obesity who are at risk for cancer or who have cancer [118]:

• Short-term weight reduction interventions may favorably affect cancer prognosis 5–10 years later.
• Preclinical studies support a beneficial effect of calorie restriction and weight loss on both chemically induced and spontaneous tumors
• Caloric restriction increases the life span of rodents and dogs and extends the life span and delayed cancer development in rhesus monkeys

The cancer-reducing benefits of weight reduction in patients with obesity somewhat mirrors the benefits found with healthful nutrition and physical activity. Weight reduction in patients with pre-obesity or obesity may:

• Reduce the risk for future cancer [20,119,120].
• Reduce cancer cell multiplication [119,121].
• Enhance cancer cell death [119,121].
• Reduce inflammation [122,123].
• Improved body metabolism with increased insulin sensitivity and lowered cancer-promoting sex hormones [118].
• Bariatric surgery may reduce the risk of cancer and cancer-related mortality [124], especially hormone-related cancers [125].

**Fig. 8:**
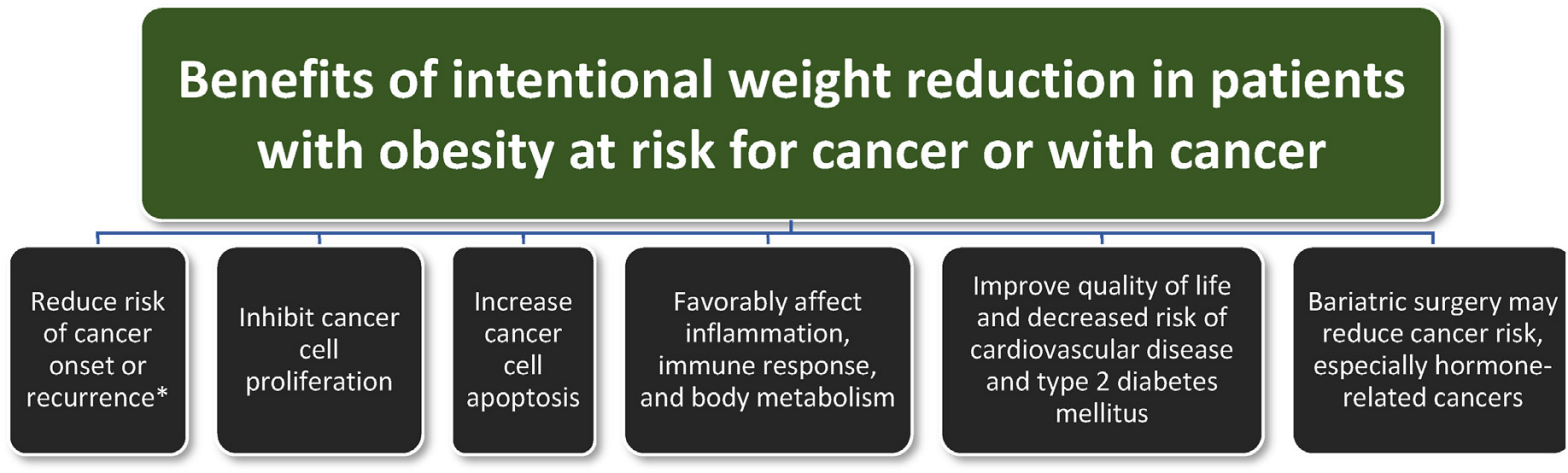
**Benefits of weight reduction in patients with cancer and obesity.** Weight reduction is beneficial for patients with pre-obesity/obesity who are at risk for cancer or who have cancer [118,121,123,124,126,127]. https://www.asco.org/sites/new-www.asco.org/files/content-files/blog-release/documents/2014-Obesity-Cancer-Guide-Oncology-Providers.pdf ∗ Potential health benefits of intentional weight loss among patients with obesity are most consistent with hormone-sensitive cancers.

Fig. 8 shows the potential benefits of weight reduction for patients with pre-obesity or obesity who are at risk of cancer or who have cancer.

## 11 Obesity and cancer: metformin

Attaining healthy body weight is a primary goal for nutritional and physical activity-based cancer management in patients with obesity. Given the adiposopathic consequences of obesity that lead to hyperinsulinemia, and given that hyperinsulinemia (a growth factor) may increase cancer risk (Fig. 4), then reducing insulin levels such as through weight reduction may help facilitate a reduced risk for cancer, and improved prognosis for existing cancer. Metformin, a biguanide medication used to treat type 2 diabetes mellitus, reduces hyperinsulinemia [128,129]. Some observational analyses suggest metformin may help reduce the overall cancer rate and help improve the treatment of multiple cancers (e.g., colon, ovary, lung, breast, and prostate cancer) [128,130,131]. However, the results of clinical intervention studies are inconsistent. If metformin does reduce cancer risk or improve cancer outcomes, then support for such a favorable effect may be best found among patients with type 2 diabetes mellitus [[132], [133], [134], [135]]. Conversely, in a study of patients with high-risk operable breast cancer without diabetes, the addition of metformin to standard breast cancer treatment did not improve invasive disease–free survival or other breast cancer outcomes [136]. These findings were consistent with other randomized trials that failed to identify a beneficial effect of metformin (in addition to standard chemotherapy or hormone therapy) on outcomes in patients with metastatic breast cancer without diabetes. All this underscores the need for well-conducted randomized trials prior to the clinical adoption of interventions that appear to benefit cancer when investigated in observational studies [136].

## 12 Obesity and cancer: ketotherapy

A dietary intervention sometimes used in management of patients with obesity is implementation of the ketogenic diet [113]. Given that cancer cells utilize glucose as their main fuel, and do not efficiently harness ketones for anabolism [137], some have suggested that a high-fat and very-low-carbohydrate diet (with adequate amounts of protein) may have antitumor effects by reducing energy supplies to cells [138] and potential effects on gene expression. While preclinical studies suggest that the ketogenic diet may have antitumor effects, prolongs survival, and prevents cancer development, human clinical trials are equivocal, and require additional study [139].

## 13 Obesity and cancer: preventative care and bias

Individuals with obesity often do not receive the same preventive standards of care as those without obesity. This can be especially relevant to certain types of cancer with increased risk associated with obesity and adiposopathy. Examples of cancer-related preventive medical care (depending upon gender and age) that patients with obesity may not adequately receive include [[140], [141], [142], [143]]:

• Breast cancer screening
• Gynecological exam with Pap smear (which may include assessment of human papilloma virus)
• Testicular and prostate cancer screening
• Colorectal cancer screening
• Skin cancer surveillance

A prior Obesity Medicine Association Clinical Practice Statement provided guidance how clinicians may address potential obesity bias within the health care setting, with practical recommendations regarding people-first language, waiting room furniture, medical equipment, and medical devices [55]. With specific regard to obesity and cancer, patients with obesity may face unique challenges in diagnostic imaging (e.g., fluoroscopy, computed tomography, and magnetic resonance imaging), with respect to weight limits, maximum aperture opening, and maximum field of view [144]. As a result of these challenges, patients at higher body mass index may not receive the same level of cancer diagnostic and imaging medical care as leaner counterparts. Whether it be cancer-related preventive medical care, or cancer-related diagnostic evaluation, ensuring patients with obesity receive appropriate and timely standards of care may help improve the prognosis of cancer among patients with obesity. The need for diligence in early detection in patients with obesity is further indicated, given that obesity-related cancers are markedly increasing among younger adults [145]. While accountability is an important component of an effective behavior modification program [146], clinicians should be mindful to avoid unproductive shame approaches to obesity and disease prevention. Cancer is not unlike other complications of obesity; blame is not a strategy [147].

## 14 Obesity and cancer: conclusions

This Obesity Medicine Association (OMA) Clinical Practice Statement (CPS) on Obesity and Cancer is designed to help clinicians manage their patients with the disease of pre-obesity/obesity. Patients with obesity are at greater risk of developing cancer. When weight reduction in patients with obesity is achieved by safe and effective interventions such as healthful nutrition and routine physical activity [101], anti-obesity pharmacotherapy [148], and bariatric surgery [149], then this may have the potential to reduce the risk of cancer, and improve the prognosis for patients who have cancer.

### Transparency [150]

This manuscript was largely derived and edited from the 2021 Obesity Medicine Association (OMA) Obesity Algorithm. Beginning in 2013, OMA created and maintained an online Adult “Obesity Algorithm” (i.e., educational slides and eBook) that underwent yearly updates by OMA authors and was reviewed and approved annually by the OMA Board of Trustees. This was followed by a similar Pediatric “Obesity Algorithm,” with updates approximately every two years by OMA authors. Authors of prior years’ version of the Obesity Algorithm are included in Supplement #1.

### Group composition

Over the years, the authors of the OMA Obesity Algorithm have represented a diverse range of clinicians, allied health professionals, clinical researchers, and academicians. (Supplement #1) The authors reflect a multidisciplinary and balanced group of experts in obesity science, patient evaluation, and clinical treatment.

## Author contributions

EL and HEB reviewed, edited, and approved the document.

## Managing disclosures and dualities of interest

Potential dualities or conflicts of interest of the authors are listed in the Individual Disclosure section. Assistance of a medical writer paid by the Obesity Medicine Association is noted in the Acknowledgements section. Neither the prior OMA Obesity Algorithms, nor the publishing of this Clinical Practice Statement received outside funding. The authors of prior OMA Obesity Algorithms never received payment for their writing, editing, and publishing work. Authors of this Clinical Practice Statement likewise received no payment for their writing, editing, and publishing work. While listed journal Editors received payment for their roles as Editors, they did not receive payment for their participation as authors.

## Individual Disclosures

EL and HEB report no disclosures.

## Evidence

The content of the OMA Obesity Algorithm and this manuscript is supported by citations, which are listed in the References section.

## Ethics review

This OMA Clinical Practice Statement manuscript was peer-reviewed and approved by the OMA Board of Trustee members prior to publication. Edits were made in response to reviewer comments and the final revised manuscript was approved by all the authors prior to publication. This submission did not involve human test subjects or volunteers.

## Conclusions and recommendations

This Clinical Practice Statement is intended to be an educational tool that incorporates the current medical science and the clinical experiences of obesity specialists. The intent is to better facilitate and improve the clinical care and management of patients with pre-obesity and obesity. This Clinical Practice Statement should not be interpreted as “rules” and/or directives regarding the medical care of an individual patient. The decision regarding the optimal care of the patient with pre-obesity and obesity is best reliant upon a patient-centered approach, managed by the clinician tasked with directing an individual treatment plan that is in the best interest of the individual patient.

## Updating

It is anticipated that sections of this Clinical Practice Statement may require future updates. The timing of such an update will depend on decisions made by Obesity Pillars Editorial team, with input from the OMA members and OMA Board of Trustees.

## Disclaimer and limitations

Both the OMA Obesity Algorithms and this Clinical Practice Statement were developed to assist health care professionals in providing care for patients with pre-obesity and obesity based upon the best available evidence. In areas regarding inconclusive or insufficient scientific evidence, the authors used their professional judgment. This Clinical Practice Statement is intended to represent the state of obesity medicine at the time of publication. Thus, this Clinical Practice Statement is not a substitute for maintaining awareness of emerging new science. Finally, decisions by practitioners to apply the principles in this Clinical Practice Statement are best made by considering local resources, individual patient circumstances, patient agreement, and knowledge of federal, state, and local laws and guidance.

## Individual Disclosures

EL and HEB report no disclosures.

## Footnotes

Appendix A Supplementary data to this article can be found online at https://doi.org/10.1016/j.obpill.2022.100026.
