# Supplementary material for: Cancer and Obesity: An Obesity Medicine Association (OMA) Clinical Practice Statement (CPS) 2022
Source: Obes Pillars. 2022 Jul 5;3:100026. doi: 10.1016/j.obpill.2022.100026 (PMC10661911; doi:10.1016/j.obpill.2022.100026)
Supplement: Multimedia component 1 [file mmc1.docx]

**Supplement #1: Historic Citation and Acknowledgement of Authorship of The Obesity Medicine Association Obesity Algorithms**

2021

eBook Citation: Bays HE, McCarthy W, Burridge K, Tondt J, Karjoo S, Christensen S, Ng J, Golden A, Davisson L, Richardson L. Obesity Algorithm eBook, presented by the Obesity Medicine Association. www.obesityalgorithm.org. 2021.. <https://obesitymedicine.org/obesity-algorithm/>

Free Slide Citation: Bays HE, McCarthy W, Burridge K, Tondt J, Karjoo S, Christensen S, Ng J, Golden A, Davisson L, Richardson L. Obesity Algorithm eBook, presented by the Obesity Medicine Association. www.obesityalgorithm.org. 2021. <https://obesitymedicine.org/obesity-algorithm-powerpoint/>

2020

eBook Citation: Bays HE, McCarthy W, Christensen S, Tondt J, Karjoo S, Davisson L, Ng J, Golden A, Burridge K, Conroy R, Wells S, Umashanker D, Afreen S, DeJesus R, Salter D, Shah N, Richardson L. Obesity Algorithm eBook, presented by the Obesity Medicine Association. www.obesityalgorithm.org. 2020. <https://obesitymedicine.org/obesity-algorithm/>

Free Slide Citation: Bays HE, McCarthy W, Christensen S, Tondt J, Karjoo S, Davisson L, Ng J, Golden A, Burridge K, Conroy R, Wells S, Umashanker D, Afreen S, DeJesus R, Salter D, Shah N, Richardson L. Obesity Algorithm Slides, presented by the Obesity Medicine Association. www.obesityalgorithm.org. 2020. <https://obesitymedicine.org/obesity-algorithm-powerpoint/>

2019

eBook Citation: Bays HE, McCarthy W, Christensen S, Wells S, Long J, Shah NN, Primack C. Obesity Algorithm eBook, presented by the Obesity Medicine Association. www.obesityalgorithm.org. 2019. <https://obesitymedicine.org/obesity-algorithm/>

Free Slide Citation: Bays HE, McCarthy W, Christensen S, Seger J, Wells S, Long J, Shah NN, Primack C. Obesity Algorithm Slides, presented by the Obesity Medicine Association. www.obesityalgorithm.org. 2019. <https://obesitymedicine.org/obesity-algorithm-powerpoint/>

2017-2018

Bays HE, Seger, J, Primack C, Long J, Shah NN, Clark TW, McCarthy W. Obesity Algorithm, presented by the Obesity Medicine Association. www.obesityalgorithm.org. 2017-2018

2016-2017

Bays HE, Seger JC, Primack C, McCarthy W, Long J, Schmidt SL, Daniel S, Horn DB, Westman EC: Obesity Algorithm, presented by the Obesity Medicine Association. www.obesityalgorithm.org. 2016-2017

2015-2016

Seger JC, Horn DB, Westman EC, Primack C, Long J, Clark T, McCarthy W, Bays HE. Obesity Algorithm, presented by the Obesity Medicine Association, 2015-2016.

2014-2015

Seger JC, Horn DB, Westman EC, Primack C, Schmidt SL, Ravasia D, McCarthy W, Ferguson U, Sabowitz BN, Scinta W, Bays HE. Obesity Algorithm, presented by the American Society of Bariatric Physicians, 2014-2015.

2013-2014

Seger JC, Horn DB, Westman EC, Lindquist R, Scinta W, Richardson LA, Primack C, Bryman DA, McCarthy W, Hendricks E, Sabowitz BN, Schmidt SL, Bays HE. Obesity Algorithm, presented by the American Society of Bariatric Physicians, 2013-2014.
